# Fast escape of a quantum walker from an integrated photonic maze

**DOI:** 10.1038/ncomms11682

**Published:** 2016-06-01

**Authors:** Filippo Caruso, Andrea Crespi, Anna Gabriella Ciriolo, Fabio Sciarrino, Roberto Osellame

**Affiliations:** 1 LENS, QSTAR & Dipartimento di Fisica e Astronomia, Università di Firenze, via Nello Carrara 1, I-50019 Sesto Fiorentino, Italy; 2 Istituto di Fotonica e Nanotecnologie, Consiglio Nazionale delle Ricerche (IFN-CNR), Piazza Leonardo da Vinci 32, I-20133 Milano, Italy; 3 Dipartimento di Fisica, Politecnico di Milano, Piazza Leonardo da Vinci 32, I-20133 Milano, Italy; 4 Dipartimento di Fisica, Sapienza Università di Roma, Piazzale Aldo Moro 5, I-00185 Roma, Italy

## Abstract

Escaping from a complex maze, by exploring different paths with several decision-making branches in order to reach the exit, has always been a very challenging and fascinating task. Wave field and quantum objects may explore a complex structure in parallel by interference effects, but without necessarily leading to more efficient transport. Here, inspired by recent observations in biological energy transport phenomena, we demonstrate how a quantum walker can efficiently reach the output of a maze by partially suppressing the presence of interference. In particular, we show theoretically an unprecedented improvement in transport efficiency for increasing maze size with respect to purely quantum and classical approaches. In addition, we investigate experimentally these hybrid transport phenomena, by mapping the maze problem in an integrated waveguide array, probed by coherent light, hence successfully testing our theoretical results. These achievements may lead towards future bio-inspired photonics technologies for more efficient transport and computation.

## Abstract (web summary)

Studying quantum transport in biological systems is difficult so developing an artificial platform that can be used to understand quantum transport is desirable. Here, Caruso, *et al*. demonstrate how a quantum walker can quickly reach the output of a maze by partially suppressing the presence of interference.

## Graphical abstract

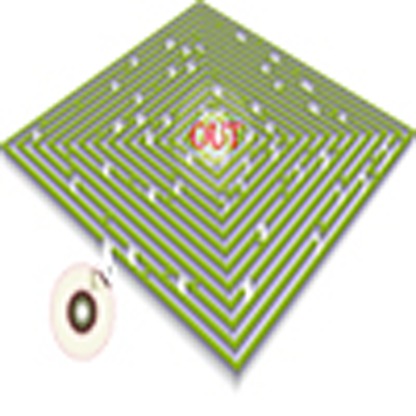

Transport problems are very popular in several fields of science, as biology, chemistry, sociology, information science, physics and even in everyday life. One of the most challenging transport problems is represented by efficiently traversing a maze, that is, finding the exit in the shortest possible time of a topologically complex network of interconnected sites (Fig. 1). The efficiency in reaching the exit of a maze dramatically decreases with the number of sites in the structure, rapidly making this problem intractable^1^.

The problem of solving mazes has fascinated mankind since the ancient times. One famous maze is the Cretan one, designed by the architect Daedalus, build to hold the mythological creature Minotaur that was eventually killed by the hero Theseus. To find the Minotaur he used the most typical maze-solving strategy: exploring several possible alternatives, while marking the visited paths (by a ball of thread). Around 60 years ago, Shannon realized the first ever experiment on maze-solving that was based on physical means, in particular an electromagnetic mouse Theseus^2^. Nowadays, the availability of new physical, chemical and biological systems has opened up the way for traversing a maze with a parallel exploration of all possible transport channels at the same time. For instance, in ref. 3 a maze is experimentally solved by filling it with a Belousov–Zhabotinsky reaction mixture and then exploiting the superposition effect of travelling chemical wavefronts. More recently, it was shown that this parallel addressing can be indirectly obtained by the chemo-attractant waves emitted by the oat flake placed at the destination site, while a plasmodium slime walks directly to the exit^4^. This demonstrates the crucial role of interference to find the maze's exit in a more efficient way.

In the framework of quantum mechanics, even a single particle, represented by a wavefunction, shows interference effects. Exploiting this property, a quantum walker is able to propagate in the fastest way inside perfectly ordered lattices^5^,^6^; however, localization phenomena may occur when disorder is present^7^,^8^,^9^,^10^. Quantum walks find applications to energy transport^11^ and quantum information^12^,^13^,^14^,^15^ with polynomial as well as exponential speedup^16^, for example, Grover search algorithm^17^, universal models for quantum computation^18^, state transfer in spin and harmonic networks^19^,^20^,^21^ and recent proposals on web page ranking^22^. Recently, the maze problem has been converted into a quantum search problem to get a quadratic speedup^23^. Interestingly enough, the interplay of interference and noise effects can further enhance quantum transport over complex networks, as recently observed for energy transport phenomena in light-harvesting proteins^24^,^25^,^26^,^27^,^28^ and proposed for noise-assisted quantum communication^29^. In particular, it is extremely difficult to study quantum transport phenomena in biological systems, as well as to change in a controlled way the problem parameters to fully understand their role. For this reason, it is very important to develop a perfectly controlled artificial platform that can be used to simulate, understand and engineer these phenomena.

In the last years, several technological platforms have been employed to investigate quantum transport phenomena, such as NMR^30^,^31^, trapped ions^32^,^33^, neutral atoms^34^ and several photonic schemes as bulk optics^35^,^36^, fibre loop configurations^37^,^38^ and miniaturized integrated waveguide circuits^39^,^40^,^41^,^42^,^43^. Among these, a very interesting experimental platform is represented by three-dimensional waveguide arrays, fabricated by femtosecond laser micromachining^41^,^44^,^45^,^46^. Femtosecond laser waveguide writing^47^ enables to fabricate high-quality optical waveguides, directly buried in the bulk of a transparent substrate. Ultrashort laser pulses are focused at the desired depth in the substrate and nonlinear absorption processes induce localized and permanent refractive index increase; translation of the sample at uniform speed allows to draw guiding paths in the substrate with unique three-dimensional design freedom. Many diverse quantum phenomena^48^,^49^ can be observed and simulated by means of such structures: in particular, a powerful analogy can be exploited between the Schrödinger equation, describing the evolution of a wavepacket in a two-dimension potential, and the equations describing the paraxial evolution of light into a dielectric structure, such as a waveguide array. In particular, an array of coupled waveguides is equivalent to a two-dimensional array of quantum wells. The temporal evolution of a single quantum particle, placed initially in a certain well, can be mapped to the spatial evolution along the propagation direction of a single photon, injected initially in a certain waveguide.

Here, we investigate the role of a partial suppression of interference effects in the transport dynamics through maze-like graphs. In particular, we theoretically demonstrate that an optimal mixing of classical and quantum dynamics leads to a remarkably efficient transmission of energy/information from the input to the exit door of a generic maze. In addition, we show that it is possible to reproduce experimentally these dynamics in a photonic simulator, unfolding the maze onto a femtosecond-laser-written three-dimensional waveguide array, where noise is implemented by modulating the propagation constants of the waveguides during the writing process. The results provide a clear demonstration that a controlled amount of decoherence in the walker can produce an enhanced transport efficiency in escaping the maze and that these phenomena can be investigated in an experimentally accessible platform and not only in abstract models.

## Results

### Theory

The maze structure is created here by the so-called random Depth-First Search algorithm applied on a square lattice of *N* nodes^50^ (see the Methods section: Maze construction, together with Supplementary Fig. 1). The transport model is represented by a walker entering the maze in some initial (IN) site or input door and moving over the structure until reaching a final (OUT) site or exit door (maze's solution).

Following the framework of quantum stochastic walks^28^,^51^, the density matrix *ρ* describing the state of the system evolves according to the Lindblad master equation:


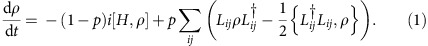


A purely unitary evolution, given by the hermitian Hamiltonian *H*, which implements the quantum walk dynamics, is mixed with an incoherent evolution describing a classical random walk, given by the operators *L*_*i*,*j*_. The balance between the two parts of the Lindblad superoperator is given by the value of the parameter *p*. In particular, for *p*=0 a fully coherent (pure interference) dynamics is observed, whereas *p*=1 corresponds to the case of classical random walk, that is, classical random hopping with no interference; for intermediate values, a mixing of the two types of behaviour is obtained. An irreversible transfer process from the exit site to an external sink is added and the walker's probability in getting the exit at time *t* is quantified by transfer efficiency function to the sink 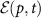, whose values are in the range (0, 1). Further technical details are given in Supplementary Note 1.

**Figure 2:**
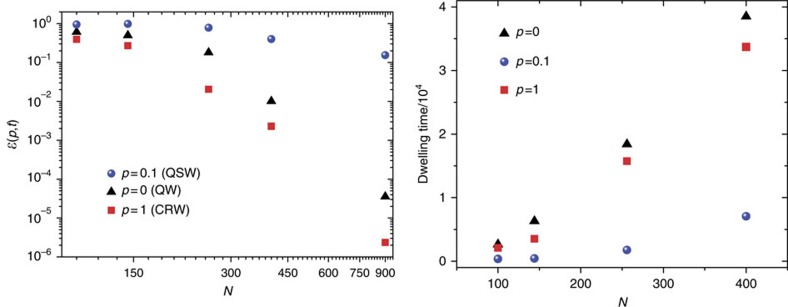
Transport efficiency for different sizes. Left: Transfer efficiency 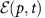 as a function of the size *N* of the maze, for a time scale *t* linearly increasing with *N*, that is, *t*=10 *N*. For a maze with *N*=900 nodes, the optimal mixing *p*≃0.1 provides a transfer efficiency that is about five orders of magnitude larger than the perfectly coherent (quantum, that is, *p*=0) and fully noisy (classical, that is, *p*=1) regimes. The trend of the curves with the maze complexity *N*, for the different values of *p*, indicates that even higher speedup can be achieved for increasingly larger mazes. Right: Dwelling time 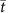 as a function of the size *N* of the maze, for *p*=0, 0.1, 1.

As shown in the left side of Fig. 2, the transfer efficiency for a maze of about one thousand sites, for a given time (linearly increasing with the maze size), is more than five order of magnitudes larger when one partially suppresses interference effects (*p*≃0.1, that is, ∼10% of mixing), with respect to the limiting cases of purely coherent and fully classical dynamics. Such transport enhancement is based on an intricate interplay between coherence and noise and shows peculiar features that makes it a fascinating field to investigate. In fact, an analogous optimal mixing has been very recently demonstrated over a large family of complex networks for *p*≃0.1 (ref. 28) and experimentally observed in ref. 52 (for the robustness of this mixing value see Supplementary Note 1 and Supplementary Fig. 2). In addition, noise-enhanced transport dynamics was observed even for totally regular and ordered graphs^53^ (where an intuitive picture of this optimality can be given in terms of a ‘momentum rejuvenation'), thus evidencing how this phenomenon cannot be explained as just a cross-over from disorder-induced coherent localization towards classic diffusive regime. As in ref. 53, we can analyse the transport inefficiency in terms of the average dwelling time in the network, which we define as 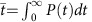, with *P*(*t*) being the population remaining on the network, that is the probability that at time *t* the energy quantum has failed to exit the network—see the right side of Fig. 2. This further supports the behaviour observed above for the transfer efficiency at long time scales (Fig. 2 left), showing that our particular choice of the time *t* for the plotted 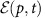 does not affect our conclusions.

One can consider how in the noiseless case the particle undergoes discrete diffraction in the structure: the strong interference effects given by full coherence generate bright and dark zones, even if the wavefunction does not strictly localize, and this may limit the transfer efficiency between two distant sites of the graph. Adding an optimal quantity of noise may help in suppressing the fine-grained interference pattern while keeping the wavefunction spread almost as in the ballistic case, without reaching the diffusive limit where the transport dynamics is much slower. Although the Lindblad model introduces decoherence only through direct classical transitions (*T*_1_-like processes), a similar behaviour would be obtained by considering a pure dephasing process (*T*_2_-like)—see (refs 27, 28, 29, 53).

### Experimental realization

**Figure 3:**
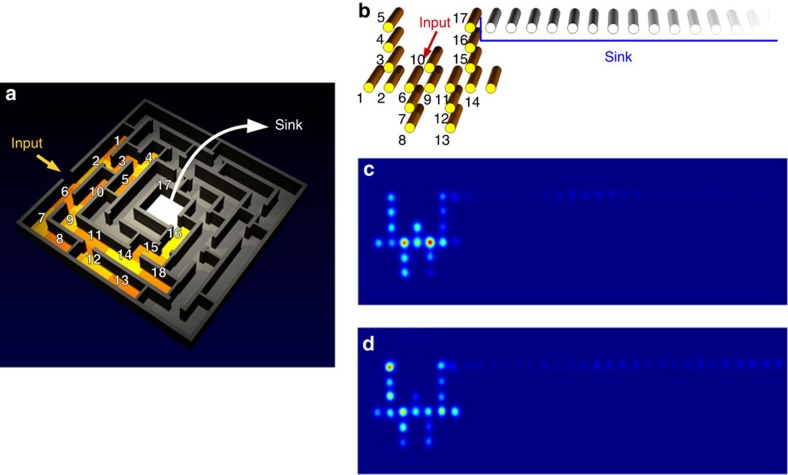
Implementing the maze. (**a**) Maze structure that is experimentally implemented; (**b**) unfolding of the maze into an almost linear graph, where each node is represented by a wave guide; (**c**,**d**) snapshots of the light diffusion for uniform (**c**) and noisy structure (**d**). The latter pictures correspond both to a propagation length of 60 mm. The noisy configuration is *noise 3* in Fig. 4.

Taking advantage of the unique three-dimensional fabrication capabilities of femtosecond laser waveguide writing, we implement a simulator of quantum stochastic walks by engineering an integrated photonic device probed by laser light. In fact, the probability distribution at the output for a single photon is perfectly reproduced by the intensity distribution of coherent light in the waveguide array. The maze structure is mapped onto a three-dimensional waveguide array, in which each waveguide represents a site of the maze. In particular, our experimental study is focused on the maze configuration shown in Fig. 3a, composed of 18 sites, taken as a significant example for observing the dynamics predicted by our theoretical model.

A first problem that has to be addressed is how to map in a waveguide system the topology of the links between the sites of our maze. Whereas in an arbitrary maze structure transfer between adjacent sites can be inhibited by walls, in waveguide arrays the coupling between two waveguides is solely determined by their relative distance. Thus, the geometry of the array needs to be engineered to keep far enough from each other waveguides that must not couple. This might not be possible if the maze graph is too complex. In our case, however, it was possible to unfold the maze graph in Fig. 3a, by considering chains with side tails, onto the partially linear and more feasible structure in Fig. 3b. Note that this unfolded geometry is not unique, other configurations being conceivable in principle with the same distances between equally coupled sites.

Another experimental issue is the realization of the exit door (that is, OUT site). In the theoretical model, this site should behave like a sink that absorbs energy irreversibly. In our photonic implementation, the sink is implemented by a long chain of waveguides (62 waveguides), which approximates well a one-way energy transfer process, with negligible probability for the light to be coupled back to the system.

Structures composed of uniform waveguides correspond to the purely coherent case (QW). Fully coherent transport dynamics in such maze can be studied straightforwardly by fabricating arrays with different lengths and characterizing the output distribution when coherent light is injected in the desired initial site (IN). It is worth noting that in this realization, the evolution parameter *t*, considered in the theoretical model, is mapped onto the propagation length, which we still label as *t*.

A controlled amount of noise is introduced in the structure by segmenting the waveguides corresponding to the sites of the maze. This is achieved by modulating the writing speed in the fabrication process, which induce a proportional variation of the propagation constant, while keeping the coupling coefficient unvaried^46^. The value of the propagation constant variation in each segment is randomly picked from a uniform distribution with a given amplitude; the same distribution is used for every waveguide within the same array. The random variation of the propagation constants is equivalent to a random variation of the site energy due to the interaction with an incoherent environment^25^, hence effectively adding also pure dephasing in the dynamics. This approach has been extensively tested by numerical simulations of this specific implementation as compared with the theoretical Lindblad model discussed in the previous section (for further details, see Supplementary Notes 2 and 3, together with Supplementary Figs 3 and 4).

The waveguide array implementing the sink is in all cases composed by uniform, not-segmented, waveguides. To characterize the transfer efficiency to the sink, the output facet of each fabricated structure is imaged onto a CMOS camera (examples of snapshots are shown in Fig. 3c,d), the light intensity on the maze and sink regions of the array are numerically integrated and the fraction of light in the sink is calculated. Technical details of the characterization procedure are given in the Methods section (Characterization measurements: experimental details).

Twenty-four structures were fabricated with the transverse layout as in Fig. 3b, implementing six different propagation lengths for both the noiseless, fully coherent, situation and three different noise configurations with the same strength (that is, same amplitude of propagation constant distribution). Waveguide arrays were inscribed in EAGLE2000 (Corning) glass substrates, by femtosecond laser writing. A Yb-based amplified laser system (FemtoREGEN, HighQLaser) was used, providing laser pulses with 400 fs duration and 300 nJ energy at 1 MHz repetition rate. The laser was focused in the substrate by a 0.45 numerical aperture, × 20 microscope objective, compensated for spherical aberrations at 170 μm below the glass surface, which is the average depth of the fabricated structures. The waveguides yield single-mode operation at the wavelength of 850 nm and the coupling coefficient between nearest-neighbouring waveguides is *κ*=0.40 mm^−1^. The amplitude of the random distribution of the propagation constants, adopted in the noise implementation, is Δ*β*_max_=0.40 mm^−1^. The modulation of the propagation constant is achieved by proportionally varying the waveguide writing speed in the 10–40 mm s^−1^ range (see also Supplementary Fig. 5 for details). In fact, varying the writing speed means changing the amount of deposited energy in the material, which, in the above range, causes a proportional variation of refractive index change and thus of Δ*β*. The value of the propagation constant is modified every 3 mm of waveguide length.

### Transfer efficiency

**Figure 4:**
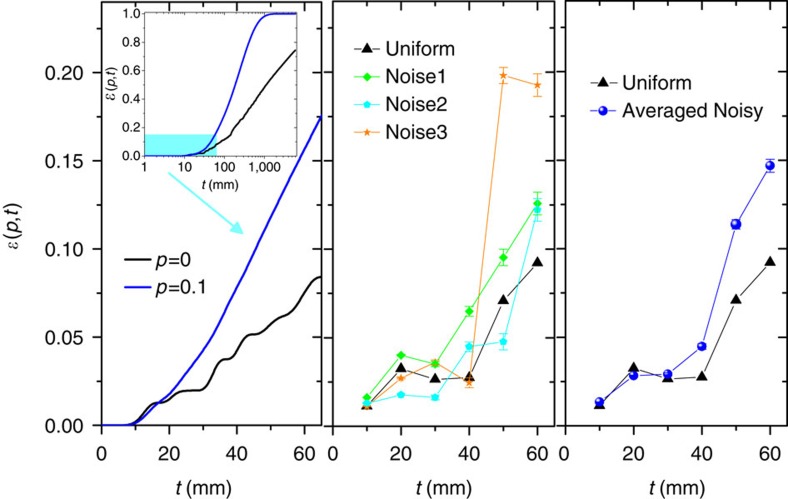
Role of noise in the transfer efficiency time evolution. Left: Theoretical behaviour of the transfer efficiency 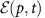 as a function of the evolution parameter *t* for two values of the mixing parameter *p*, corresponding to QW (*p*=0) and QSW (*p*=0.1), for the maze in Fig. 3. Inset: a larger time scale is shown in order to point out the remarkable efficiency enhancement when there is a partial suppression of interference. Centre: Experimental results for both uniform (triangles) and three noisy realizations of the structure reported in Fig. 3. Where not shown the error bars are smaller than the mark size. Right: as in the middle panel, but only with the uniform case (triangles) and with the average efficiency over the noise realizations (circles). In all three panels, the time *t* is in units of mm because it is experimentally mapped into the propagation length of the three-dimensional waveguide array.

The transfer efficiency to the sink, calculated theoretically with the method reported in ref. 28, is shown in Fig. 4 for a maze with the layout presented in Fig. 3. Such layout represents the actual structure that we have experimentally implemented and is considered both for the case of fully coherent quantum transport and for the case of partially incoherent transport with *p*=0.1. Figure 4 shows the experimentally retrieved transfer efficiencies, each point corresponding to a physically different structure fabricated to implement a certain noise map and a given propagation length. The average between the points corresponding to the three different noise implementations is also shown (right panel).

The physical quantity that is measured experimentally is the fraction of light present in the sink after a certain propagation, and not the fraction of light that is transferred to the sink. In case propagation losses are the same both in the maze waveguides and in the sink waveguides, the two quantities indeed correspond. As a matter of fact, the modulation of the writing speed produces additional losses in the waveguides of the maze with respect to the waveguides of the sink and this causes in general an overestimation of the transfer efficiency. However, we characterized accurately such additional losses in our structures and simulated their impact on the estimation of the transfer efficiency. The consequent systematic error contribution has been directly taken into account in Fig. 4, whereas the random error contribution is reported with the error bars. Details on the treatment of noise and error contributions in the efficiency estimation are given in the Methods section (Characterization measurements: experimental details).

A very good agreement between theoretical and experimental curves is observed both for the noiseless, fully coherent case, and for the partially coherent transport when considering the average of our ‘noisy' waveguide implementations. Therefore, this experimental evidence is consistent with our claim that the interplay of noise and interference effects leads to higher efficiency in finding the way out from the maze.

**Figure 5:**
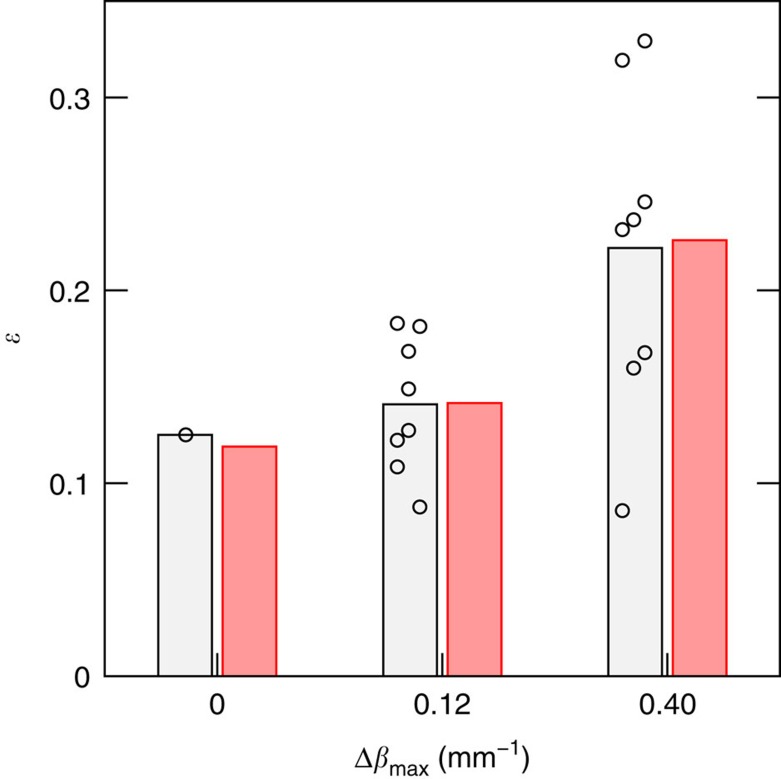
Averaging over different noise realizations. Experimentally measured transfer efficiency in photonic maze structures with the topology of Fig. 3 and propagation length *t*=60 mm. Both uniform and different noise maps with Δ*β*_max_=0.12 mm^−1^ and Δ*β*_max_=0.40 mm^−1^ have been implemented. The light grey (left) bars indicate the average over several experimental results obtained for different noise maps with the same amplitude Δ*β*_max_ (single measurements are reported as open circles). The red (right) bars represent the expected average transfer efficiency, numerically simulated for 1,000 different noise maps with the given Δ*β*_max_. Error bars are smaller than the mark size.

To further assess our experimental observation of a noise-induced enhancement in transfer efficiency in this platform, we fabricated other photonic structures implementing the maze of Fig. 3 at an evolution parameter *t*=60 mm, but employing a different femtosecond laser writing setup for the fabrication (see Supplementary Note 4 for details). We fabricated one uniform structure and 16 different random ‘noisy' implementations, 8 with a noise strength Δ*β*_*max*_=0.12 mm^−1^ and 8 implementing a noise strength Δ*β*_max_=0.40 mm^−1^. As shown in Fig. 5, we can experimentally observe a transfer efficiency of 12.5% for the uniform case, to be compared with an average transfer efficiency of 14.1% and 22.2% for the Δ*β*_max_=0.12 mm^−1^ and Δ*β*_max_=0.40 mm^−1^ cases, respectively. The experimental data are in good agreement with the simulations, which take into account small differences in the waveguide properties with respect to those fabricated with the previous system. It can be noticed that the distribution of the measurements (open circles in Fig. 5) around the mean value spreads with increasing disorder: however, the increase in the average transfer efficiency is clear when the amount of noise approaches the optimum value.

## Discussion

To summarize, here we have studied both theoretically and experimentally the dynamics of a walker travelling in a maze, having a single path from the input door (starting point) to the exit (solution). By considering a model that mixes the behaviour of a classical walker and a quantum one, we have found an optimal condition leading to extremely efficient and fast transmission. For large enough maze size, this leads to a remarkably high enhancement of more than five order of magnitudes in the transfer efficiency with respect to both the classical and purely quantum limits. This result is a clear example that decoherence is not always a detrimental phenomenon that should be avoided in quantum processes and it may provide some insight on the reason why nature evolution has made the observation of purely coherent phenomena so difficult.

By exploiting the unique capabilities of the femtosecond laser writing technology, we have unfolded the maze and implemented it in a three-dimensional waveguide array, where a suitable modulation of the waveguide properties allowed us to mimic a partial decoherence of the walker. Our measurements have faithfully confirmed the theoretical predictions and, in particular, the remarkable role of a partial suppression of interference in enhancing transport dynamics in mazes. It is also worth noting that our technological platform has enabled an experimental simulation of a noise-assisted problem in well-controlled conditions and over complex topologies, and can thus represent a very powerful tool for further studies in this direction. These results, together with future full circuit reconfigurability, will pave the way to much more complex integrated photonics devices exploiting interference, quantum features and noise effects for improved problem-solving efficiency, and remarkably fast transmission of information in ICT applications and of energy in novel solar technologies.

Another experimental demonstration of enhanced quantum transport by controlled decoherence has been reported during the preparation of this manuscript^54^.

## Methods

### Maze construction

Depth-First Search algorithm is the simplest maze generation algorithm and is based on the following iterative procedure that is applied to a regular square grid of *N* nodes, where all neighbour sites are separated by a wall^50^. One starts from a random node and then search for a random neighbour that has not considered yet. If so, the wall between these two sites is knocked down; otherwise, one backs up to the previous node. This procedure is repeated until all sites of the grid have been visited. By doing so, the final structure is a maze where we have only one path connecting the IN to the OUT node, that is, a so-called two-dimensional perfect maze with no closed loops. Applying this procedure to larger and larger square lattices, one obtains increasing large maze graphs (see Supplementary Fig. 1 for details).

### Characterization measurements: experimental details

The fabricated structures are probed by coherent light. Laser light at 850 nm wavelength is injected into the input waveguide. The output distribution is imaged onto a CMOS camera by means of a 0.12 numerical aperture objective. Numerical integration on different parts of the acquired image allows to retrieve the fraction of light present in the sink waveguide array. The advantages of this method are, on one hand, insensitivity to coupling losses of the input beam and, on the other hand, the possibility of a fast acquisition of the output of many waveguides.

A careful analysis of the measurement error has been performed. A possible source of error is the quantization of the intensity levels of the CMOS sensor, as well as its finite spatial resolution. To analyse this error contribution, we simulated the numerical integration of gaussian modes, with the same size as the measured ones, but random intensity and peak position, discretized both in the (256) intensity levels and in the pixels of the spatial profile. Supplementary Fig. 6 shows the (normalized) difference between the numerically calculated integral and the analytic integral of the gaussian profile, for 1,000 randomly distributed modes, as a function of the peak intensity. Note that, given a certain peak intensity, the numerical integral may give different values depending on the position of the peak, because of pixel discretization. Systematic and random errors are almost independent on the peak intensity and they have been taken into account in data elaboration assuming that the acquired image contains *n* modes of random uniformly distributed intensity. As a matter of fact, the contribution of such errors on the experimentally measured efficiencies is relevant only for the shortest lengths, where the light intensity in the sink is particularly low.

Furthermore, cascading waveguide segments with different fabrication speed (to mimic noise) causes small additional losses, at each interface between different waveguide segments. Importantly, these losses are present only in the waveguides representing the maze and not in the sink waveguides. Because of those losses, measuring the fraction of power *present* in the sink after a certain propagation distance, with respect to the overall output power, as is done in our characterization process, may not correspond precisely to the fraction of input power *transferred* to the sink. In fact, the transfer efficiency is slightly overestimated.

Overall additional losses can be measured quite accurately, by simply measuring and comparing the insertion losses of the fabricated devices (ration of the overall output power over the input power). However, the precise contribution of these losses on each measured transfer efficiency can be hardly retrieved. In fact, the transfer process is not uniform during the propagation and depends on the *random* noise map implemented.

Thus, to statistically quantify such overestimation, we numerically simulated the light propagation in waveguide structures analogous to the fabricated ones, for 100 random different noise distributions (with always the same amplitude, as the one adopted in the experiment), both in the case of waveguides with no losses (which corresponds to the ideal situation) and in the case of waveguides yielding uniform additional losses with respect to the waveguides of the sink, in such a way that the overall losses of the structure correspond to the experimentally measured ones (∼2 dB additional losses for the longest arrays). We evaluated in each case the estimation error of the transfer efficiency and calculated the error statistical distribution. Supplementary Fig. 7 shows the average error, together with its standard deviation, as a function of the propagation length. The effect of these losses reveals to be small (the systematic component is <3% for 60 mm, correspondent to our longest fabricated devices) and does not significantly influence our experimental observation of an increase in transfer efficiency in the cases in which noise is added.

The data that support the findings of this study are available from the corresponding authors upon request.

## Additional information

**How to cite this article:** Caruso, F. *et al*. Fast escape of a quantum walker from an integrated photonic maze. *Nat. Commun.* 7:11682 doi: 10.1038/ncomms11682 (2016).

## Supplementary Material

Supplementary Information Supplementary Figures 1-7, Supplementary Notes 1-4 and Supplementary References.

## Figures and Tables

**Figure 1:**
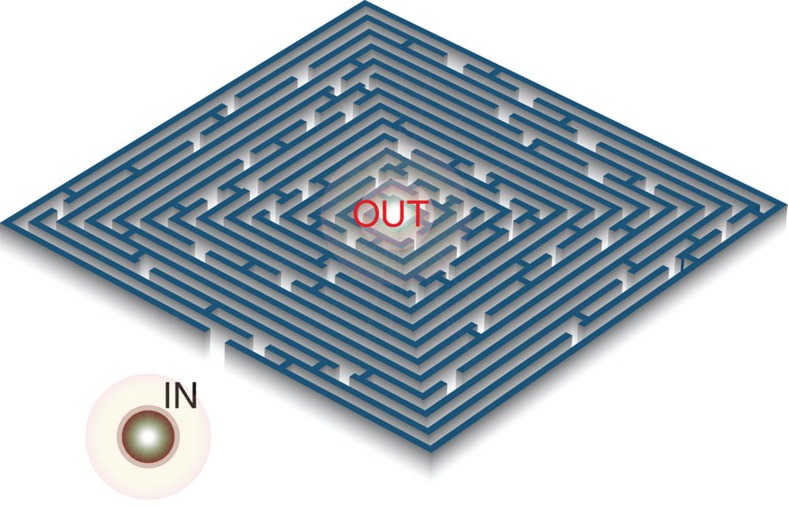
Maze problem. Pictorial view of a maze with single input (IN) and output (OUT) ports. An ideal walker has to travel from IN to OUT in the shortest possible time.
