# Supplementary material for: Fast escape of a quantum walker from an integrated photonic maze
Source: Nat Commun. 2016 Jun 1;7:11682. doi: 10.1038/ncomms11682 (PMC4895438; doi:10.1038/ncomms11682)
Supplement: Supplementary Information — Supplementary Figures 1-7, Supplementary Notes 1-4 and Supplementary References. [file ncomms11682-s1.pdf]

Supplementary Figures

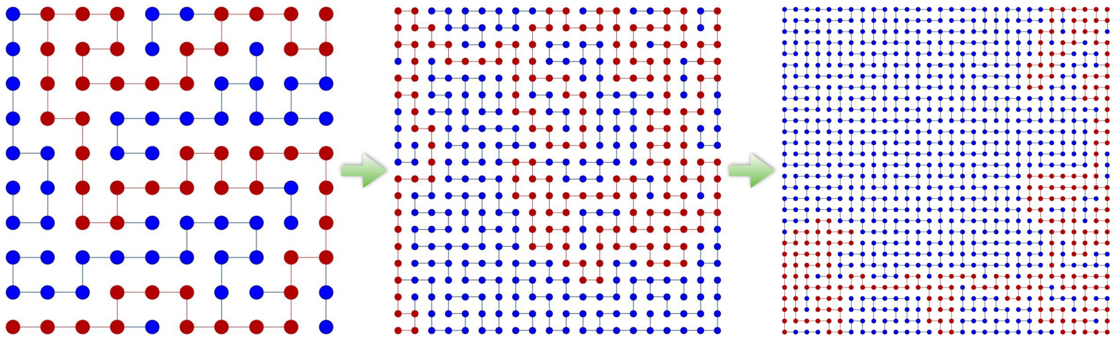

Supplementary Figure 1: Examples of mazes of different size. Making the maze larger and larger by applying DFS algorithm to a square lattice of higher number N of nodes. The only pathway connecting the IN and OUT sites (placed at the opposite corners of the lattice) is shown in red.

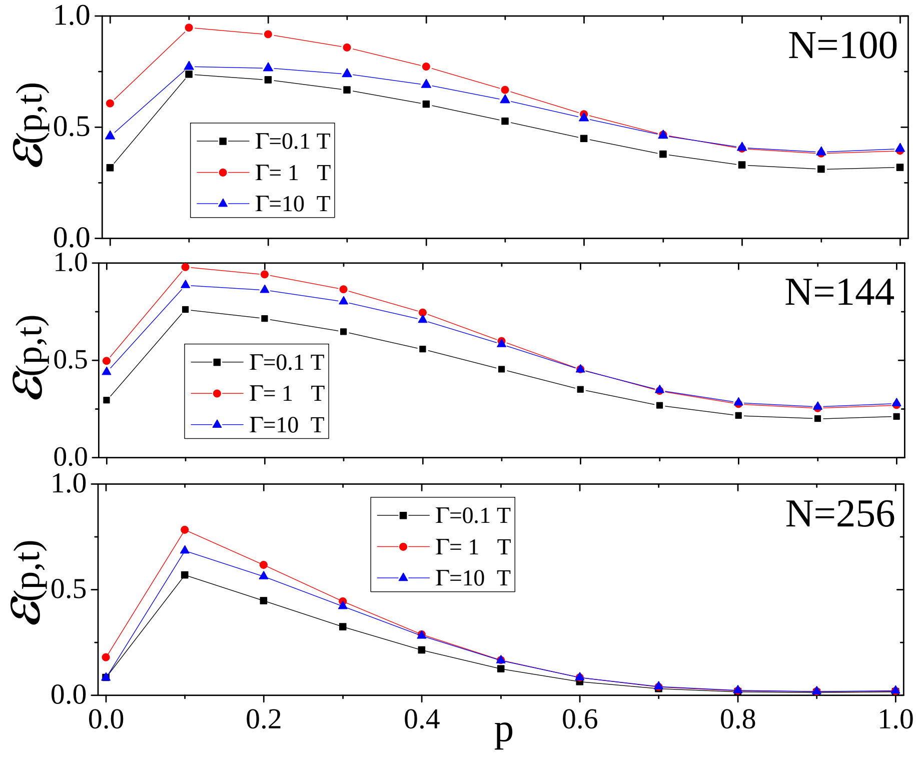

Supplementary Figure 2: Influence of the mixing parameter p. Transfer efficiency $\mathcal{E}(p,t)$ as a function of p, for a time scale t linearly increasing with N, i.e. $t = 10\ N$, for mazes of size $N = 100$, $N = 144$ and $N = 256$ (from top to bottom). In each panel the transfer rate Γ is varied by two order of magnitudes in order to show the robustness of our results with respect to the specific choice of Γ.

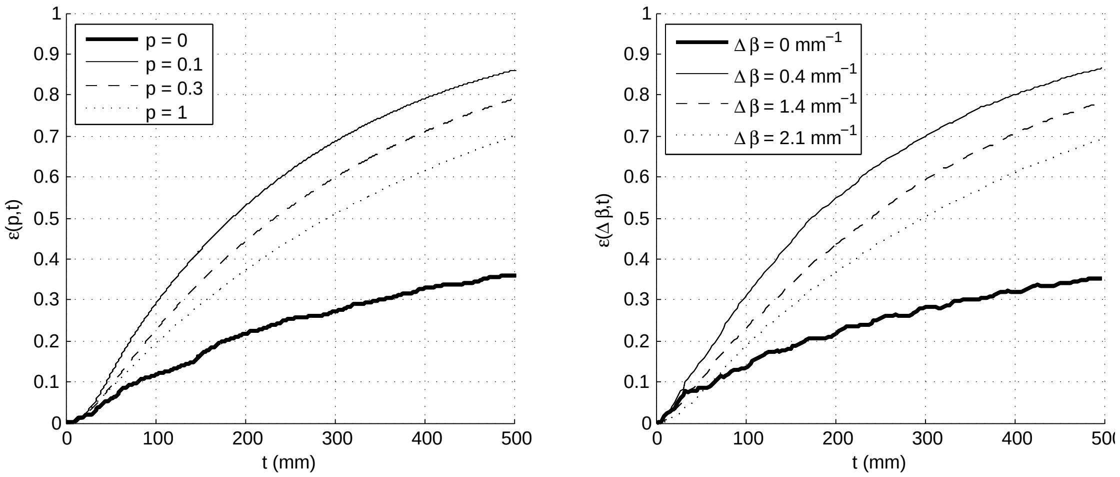

Supplementary Figure 3: Comparison of the Lindblad model vs photonic model. Transfer efficiency as a function of the propagation length t, calculated with a quantum stochastic walk model for different values of p (left), and with a photonic simulation for different amplitudes of the random distribution of $\Delta\beta$ (right). For the photonic simulation the average over 100 random distributions of $\Delta\beta$ with the same amplitude is shown.

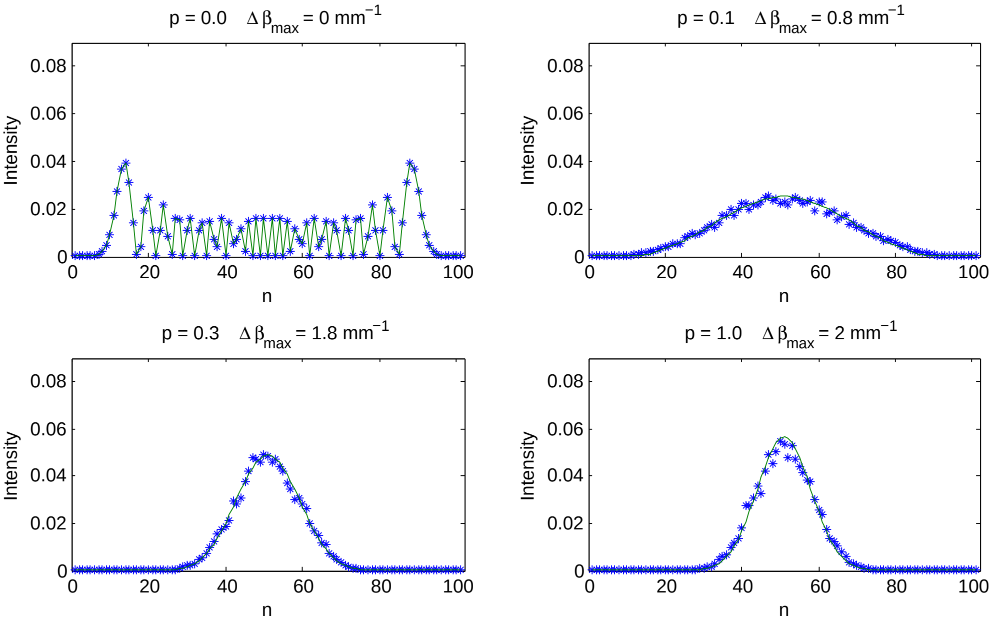

Supplementary Figure 4: Simulation of linear arrays. (stars) Simulated averaged output distribution for linear waveguide arrays of 101 waveguides, after 50 mm propagation. Propagation constants of the waveguides are modulated randomly in segments of 3 mm length, picking the values from uniform distributions of amplitude $\Delta\beta$. The coupling coefficient between adjacent waveguides is 0.4 mm^{-1}. The average is performed on 200 noise realizations. (continuous line) Simulation with the Lindblad model for an equivalent coupling between the sites and different values of p.

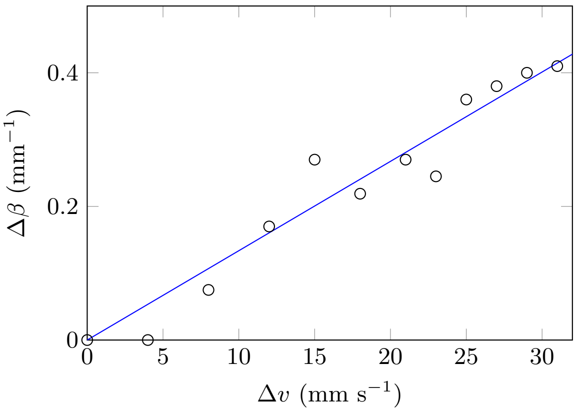

Supplementary Figure 5: Calibration of the waveguide fabrication velocity. Experimental calibration of the variation in the femtosecond-laser-written waveguide propagation constant $\Delta\beta$, as a function of the variation in the translation speed Δv, with respect to a reference waveguide written at $v = 40$ mm s $^{-1}$. The fitted linear relation is $\Delta\beta \simeq 0.013$ s mm$^{-2}\Delta v$. The measurement was performed by fabricating pairs of coupled waveguides (with known coupling coefficient), one with writing speed $v = 40$ mm s $^{-1}$ and the other with speed $v - \Delta v$; the corresponding $\Delta\beta$ was retrieved from the distribution of the light in the waveguides after a fixed propagation length[1].

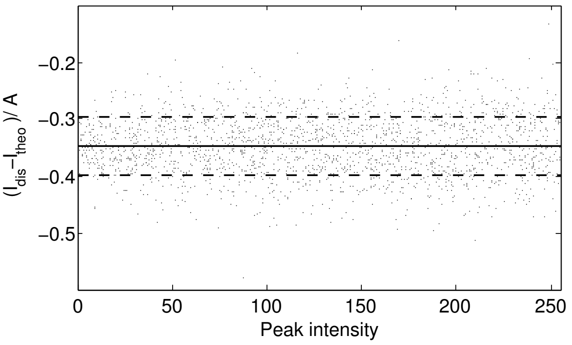

Supplementary Figure 6: Discretization error in image acquisitions. Difference between numerically calculated integral I_{dis}, affected by pixel and level discretization, and the analytical integral I_{theo} of 1000 gaussian modes with random intensity and position, normalized to the mode area $A = \pi w_x w_y$ (w_x and w_y are the 1/e mode radii in the two dimensions). The continuous horizontal line marks the average value (∼-0.347). The two dashed lines indicate the standard deviation levels ($\sim -0.347 \pm 0.052$). Average and standard deviation values have been computed on 10^5 points.

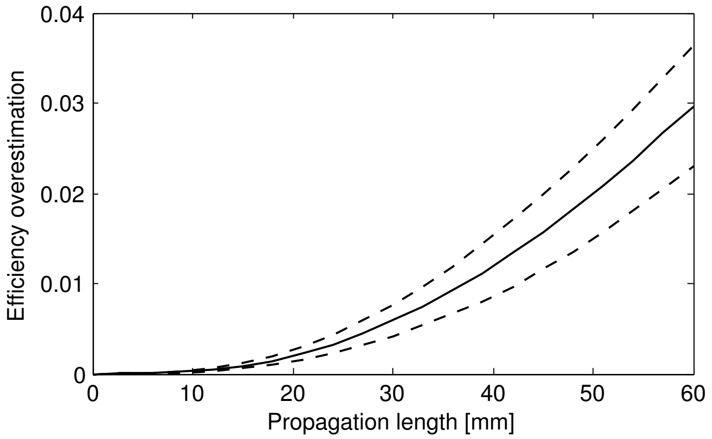

Supplementary Figure 7: Overestimation error in the measured transfer efficiency. The transfer efficiency is measured as the ration between the light present in the sink over the total output light, as a function of the propagation length. Overestimation errors arise from additional losses in the waveguides of the maze. The continuous line shows the average overestimation; the dashed lines indicate the standard deviation. The overestimation is evaluated from numerical simulations on 100 random noise distributions, with amplitude $\Delta\beta = 0.4$ mm^{-1}, where uniform additional losses are added in the waveguides of the maze.

Supplementary Notes

Supplementary Note 1 - Theoretical model for quantum stochastic walks

The analysis of the transport dynamics on the maze structure is theoretically supported by the the general framework of quantum stochastic walks [2, 3], where the physical state of the system is described by the density matrix ρ and follows this evolution:

$$\frac{d\rho}{dt} = -(1-p)i[H,\rho] + p\sum_{ij}\left(L_{ij}\rho L_{ij}^{\dagger} - \frac{1}{2}\{L_{ij}^{\dagger}L_{ij},\rho\}\right) \ , \tag{1}$$

where H is the Hamiltonian including the hopping parameters T_{ij} between the connected nodes i and j described by the states $|i\rangle$ and $|j\rangle$ (all having the same energy), while the operators $L_{ij} = T_{ij}|i\rangle\langle j|$ are responsible for the presence of the effective dephasing noise experimentally induced by dynamical disorder on the integrated structure. The limit $p = 0$ corresponds to the case of 'no noise", while $p = 1$ is obtained when the interference effects are completely destroyed and the hopping is just classical. For other values of p between 0 and 1 there is a mixing of the two types of dynamics, i.e. combining classical and quantum effects.

The transfer efficiency of the structure is measured by adding, on the right-side of Eq. 1, another term $L_{N+1} = \Gamma\ [\ 2|N+1\rangle\langle N|\ \rho\ |N\rangle\langle N+1| - \{|N\rangle\langle N|,\ \rho\}]$, with Γ being the irreversible transfer rate from the site N of the graph into some external node $N+1$ (exit door or OUT site) where the energy is continuously and irreversibly stored. Hence, the transfer efficiency of the maze will be measured by [3, 4]

$$\mathcal{E}(p,t) = 2\Gamma\int_0^t \rho_{NN}(p,t')\mathrm{d}t' \ . \tag{2}$$

where $\rho_{NN}(p,t')$ is the population of the site N at time t' in the case of a mixing strength p.

In this manuscript the model parameters are: 1) uniform nearest-neighbor couplings, i.e. $T_{ij} \equiv T$ for any connected nodes i and j, 2) next-nearest neighbor couplings between the sites in the main chain and the closest ones on the relative tails $T_{ij} \equiv 0.2\ T$ (these are sites placed at 45°, at a relative distance $\sqrt{2}$ times larger than the first neighbors), to match with the experimental observations, 3) $\Gamma = T$, 4) the time is measured in units of T^{-1}.

Let us point out that the optimality of $p \simeq 0.1$ does not depend on the specific choice of Γ. Indeed, by changing Γ by two orders of magnitudes, i.e. in the range $[0.1, 10]\ T$, the optimal transport efficiency is still at $p \simeq 0.1$ (see Supplementary Fig. 2). Notice that, as analytically and numerically shown in Ref. [5], the optimality of $p \simeq 0.1$ is quite robust but slightly move to smaller values for larger N since the transfer efficiency is inversely proportional to the network size.

We note that in the model above we have a mixing of classical hopping process and quantum walks, leading to the suppression of interference effects, that is instead experimentally implemented by means of a qualitatively different process, i.e. introducing dynamical disorder in the waveguide structure along the propagation length. However, this does not affect our results, because, by numerically considering the presence of dynamical disorder (instead of the mixing p component), we find a very similar transport efficiency behaviour (for more details see also Supplementary Note 2 and Supplementary Fig. 3).

Supplementary Note 2 - Photonic model

Before fabricating the waveguide devices, we investigated accurately by numerical simulations the possibility to implement quantum walks with mixtures of quantum and classical behaviours by our photonic structures.

Mazes with different sizes and topologies were considered. First they were unfolded as described in the Main Text, to give the geometry of feasible photonic structures. A long waveguide chain was added, coupled to the output site, to implement the sink. The propagation of coherent light, injected initially in a chosen input site, was simulated by coupled mode equations of the kind [6]:

$$i\frac{dA_n}{dz} = \Delta\beta(z)A_n + \sum_{m\neq n} \kappa_{m,n}A_m \tag{1}$$

where A_n is the field amplitude on the n-th mode, $\Delta\beta(z)$ is the detuning of the propagation constant of the waveguide, which varies randomly in segments along z to implement noise, $\kappa_{m,n}$ is the coupling coefficient between the m-th and the n-th waveguides. Coupling coefficients between third-neighbour waveguides and above were considered negligible. The transfer efficiency of the maze was calculated as the fraction of power being in the sink waveguide array.

The results of this kind of numeric simulation were compared with the behaviour predicted by the quantum stochastic walk model described above. An excellent agreement between the two procedures was evidenced: the behaviour obtained for a certain value of the parameter p of the quantum stochastic walk can be faithfully reproduced with a proper amplitude of the random distribution of $\Delta\beta$ in the photonic model. As an example, Supplementary Fig. 3 reports the transfer efficiency obtained with a quantum stochastic walk model and with the coupled-mode equations described above, for a structure with the topology of Fig. 3 of the Main Text.

Supplementary Note 3 - Mimicking decoherence

The Lindblad model discussed in the first section and described by Eq. 1 is a model which generates an interplay between a quantum walk and a classical random walk with a proportion given by the phenomenological parameter p. While the quantum-walk part of the Lindblad superoperator connects the *population* (diagonal) terms of the density matrix with the *coherence* (off-diagonal) terms, the classical part connects directly the population terms one to the other. Given an initial pure quantum state, if only the quantum-walk part were present, it would remain a pure state through the propagation; in contrast, the classical part makes it a partially mixed state.

It should be noted that the classical part in the Eq. 1 does not refer to a particular microscopic process: in contrast, in a real physical system the interaction with the environment will have its peculiar microscopic characteristics. In our realization, we aim at implementing an interaction with the environment that effectively varies the energy of the sites composing the maze, with random variations around a "zero" level (similarly also to Ref. [7]). Since the environment can be seen as an incoherent system, such random variations are purely *classical*, modelled with actual variations of the energy of the sites in specific noise realizations. Thus, as discussed in the Main Text and in Supplementary Note 2, a controlled amount of decoherence in the walk is mimicked by noise in the propagation constant; i.e., a waveguide is composed of adjacent segments of fixed length L with different propagation constants (uniform inside each segment) with values randomly picked from a uniform distribution with a given amplitude $\Delta\beta_{max}$.

Note that at the end of each segment and at the end of the propagation, the specific noise map implemented (which is random in a classical sense) determines the pure state that we measure in the end. The population terms of such pure state are characterised with our measurements with classical light. To reconstruct the full mixed state a rich statistics of noise realizations would be needed. However agreement with the Lindblad model (which does not refer to single realization but general statistical properties of the ensemble) can be checked with a small number of realizations.

The possibility to use the Lindblad formalism for describing this kind of microscopic dynamics implemented in detail by a waveguide array with random variations of the propagation constants, or, conversely, the possibility to use such a kind of photonic system to experimentally investigate the dynamics of the Lindblad model, is further confirmed from the data in Supplementary Fig. 4. The figure shows the simulated output distributions (averaged on 200 noise realizations) after 50 mm propagation in linear arrays of 101 waveguides, where the propagation constants are modulated randomly as described above and coherent light is injected in the central waveguide, compared to simulations for different values of p for the Lindblad model. Increasing the modulation amplitude the distribution progressively change from the typical ballistic spread of the quantum walk to the gaussian shape of the classical random walk, with very good agreement between the two models.

It is interesting to note that even in such a completely ordered system, with no localized eigenstates in absence of noise, when analyzing the transport dynamics from the center of the array to one end, noise-assisted transport was recently observed for similar optimal noise conditions $p = 0.1$ [3]. As shown in Supplementary Fig. 4 the distribution for $p = 0.1$ yields marked differences with respect to both the classical ($p = 1$) and quantum ($p = 0$) limiting cases, which further emphasizes the peculiarity of this phenomenon.

While numerically, as in these simulations, an arbitrary amount of decoherence can be induced by properly increasing the modulation amplitude of the propagation constant, the experimental implementation could meet some limitations. In fact, referring for instance to the femtosecond laser writing technology adopted in this work, a too deep modulation of the writing speed (in

order to vary the propagation constant) may imply as a side effect a relevant change in other waveguide characteristics such as mode size or first- and second-neighbor coupling coefficients, thus making the structures not comparable to those uniform or with lower noise level.

Supplementary Note 4 - Complementary measurements: experimental details

To strengthen our experimental observations of an enhancement in the transfer efficiency in "noisy" mazes, we fabricated additional photonic structures, with the same geometry and layout of the ones presented in Fig. 3 of the Main text, but employing a different femtosecond laser writing source. In particular a Yb:KYW mode-locked cavity-dumped oscillator was used, which produces pulses of about 300 fs duration at 1 MHz repetition rate and 1030 nm wavelength. The adopted irradiation parameters were 20× focusing objective, 300 mW power and 40 mm/s translation speed, which produced single-mode waveguides at 850 nm.

We fabricated one uniform structure and 16 noisy ones, with different noise maps. All the structures have a propagation length $t = 60$ mm. The amplitude of the random distributions of the propagation constants, adopted in the noise implementations, is $\Delta\beta_{max} = 0.12$ mm^{-1} for 8 structures and $\Delta\beta_{max} = 0.40$ mm^{-1} for other 8 structures. Figure 5 in the Main Text reports the experimental data, compared to the results of numerical simulations, averaged on 1000 noise realizations. The numerical simulations consider light propagation in the waveguides and are performed by coupled mode equations. Small differences in the optical properties with respect to the waveguides fabricated with the previous system (in particular an increased nearest-neighbor coupling coefficient $\kappa = 0.54$ mm^{-1}) were taken into account. In these structures we did not notice any relevant additional loss in the "noisy" case with respect to the uniform one; this produced a lower uncertainty in the efficiency estimation with respect to the graph in Figure 4 of the Main Text. In fact, error bars are not shown in Figure 5 because the uncertainty is smaller or comparable to the mark size.

Supplementary References

[1] Yariv, A. Coupled-mode theory for guided-wave optics *IEEE J. Quant. Electron.* **9**, 919-933 (1973).

[2] Whitfield, J.D., Rodríguez-Rosario, C.A. & Aspuru-Guzik, A. Quantum stochastic walks: A generalization of classical random walks and quantum walks. *Phys. Rev. E* **81**, 022323 (2010).

[3] Caruso, F. Universally optimal noisy quantum walks on complex networks. New. J. Phys. **16**, 055015 (2014).

[4] Caruso, F., Chin, A.W., Datta, A., Huelga, S.F. & Plenio, M.B. Highly efficient energy excitation transfer in light-harvesting complexes: The fundamental role of noise-assisted transport. *J. Chem. Phys.* **131**, 105106 (2009).

[5] Li, Y., Caruso, F., Gauger, E. & Benjamin, S.C. Momentum rejuvenation underlies the phenomenon of noise-assisted quantum energy flow. *New J. Phys.* **17**, 013057 (2015).

[6] Perets, H.B. *et al.* Realization of Quantum Walks with Negligible Decoherence in Waveguide Lattices.*Phys. Rev. Lett.* **100**, 170506 (2008).

[7] Mohseni, M., Rebentrost, P., Lloyd, S. & Aspuru-Guzik, A. Environment-assisted quantum walks in photosynthetic energy transfer. *J. Chem. Phys.* **129**, 174106 (2008).
